# Multiplex array analysis of circulating cytokines and chemokines in COVID-19 patients during the first wave of the SARS-CoV-2 pandemic in Milan, Italy

**DOI:** 10.1186/s12865-024-00641-z

**Published:** 2024-07-26

**Authors:** Estefanía Calvo-Alvarez, Sarah D’Alessandro, Nunzia Zanotta, Nicoletta Basilico, Silvia Parapini, Lucia Signorini, Federica Perego, Kevin Kamau Maina, Pasquale Ferrante, Annalisa Modenese, Pierluigi Pizzocri, Andrea Ronsivalle, Serena Delbue, Manola Comar

**Affiliations:** 1 Department of Pharmacological and Biomedical Sciences, Università degli Studi di Milano, Via Carlo Pascal, 36, Milano, 20133 Italy; 2 Department of Advanced Translational Microbiology, Institute for Maternal and Child Health—IRCCS Burlo Garofolo, Via dell’Istria, 65, Trieste, 34137 Italy; 3 Department of Biomedical, Surgical and Dental Sciences, Università degli Studi di Milano, Via Carlo Pascal, 36, Milano, 20133 Italy; 4 Department of Biomedical Sciences for Health, Università degli Studi di Milano, Via Carlo Pascal, 36, Milano, 20133 Italy; 5 Istituto Clinico Città Studi, Via Ampere, 47, Milano, 20133 Italy

**Keywords:** COVID-19, SARS-CoV-2, Primarily-infected subjects, Serum cytokine/chemokines levels, Anti-SARS-CoV-2 IgG

## Abstract

**Background:**

The systemic inflammatory syndrome called “cytokine storm” has been described in COVID-19 pathogenesis, contributing to disease severity. The analysis of cytokine and chemokine levels in the blood of 21 SARS-CoV-2 positive patients throughout the phases of the pandemic has been studied to understand immune response dysregulation and identify potential disease biomarkers for new treatments. The present work reports the cytokine and chemokine levels in sera from a small cohort of individuals primarily infected with SARS-CoV-2 during the first wave of the COVID-19 pandemic in Milan (Italy).

**Results:**

Among the 27 cytokines and chemokines investigated, a significant higher expression of Interleukin-9 (IL-9), IP-10 (CXCL10), MCP-1 (CCL2) and RANTES (CCL-5) in infected patients compared to uninfected subjects was observed. When the change in cytokine/chemokine levels was monitored over time, from the hospitalization day to discharge, only IL-6 and IP-10 showed a significant decrease. Consistent with these findings, a significant negative correlation was observed between IP-10 and anti-Spike IgG antibodies in infected individuals. In contrast, IL-17 was positively correlated with the production of IgG against SARS-CoV-2.

**Conclusions:**

The cytokine storm and the modulation of cytokine levels by SARS-CoV-2 infection are hallmarks of COVID-19. The current global immunity profile largely stems from widespread vaccination campaigns and previous infection exposures. Consequently, the immunological features and dynamic cytokine profiles of non-vaccinated and primarily-infected subjects reported here provide novel insights into the inflammatory immune landscape in the context of SARS-CoV-2 infection, and offer valuable knowledge for addressing future viral infections and the development of novel treatments.

**Supplementary Information:**

The online version contains supplementary material available at 10.1186/s12865-024-00641-z.

## Background

Coronavirus disease 2019 (COVID-19), the disease associated with the infection with SARS-CoV-2, an enveloped, positive-sense, single-stranded RNA virus, emerged in China in 2019 and rapidly evolved into a global pandemic [1]. In Italy, the first positive case appeared on 31^st^ January 2020. Subsequently, clusters of new cases emerged in many cities of Lombardy and Veneto (two regions in the north of Italy), and then disseminated over the whole country [2], culminating with the first national lockdown between March and April 2020. Since then, more than 26.9 million cases have been reported in Italy, with a cumulative case fatality rate (CFR) of 0,7% (https://www.epicentro.iss.it/coronavirus/sars-cov-2-dashboard).

The clinical presentation of SARS-CoV-2 infection is widely variable, ranging from asymptomatic and mild cases to severe pneumonia and death [3]. The virus primarily targets human cells through the angiotensin-converting enzyme-related carboxypeptidase 2 (ACE-2) receptor, abundantly expressed in cardiopulmonary tissues, endothelial cells and in some hematopoietic cells including immune cells [4, 5]. The virus then spreads through the respiratory tract, causing viral pneumonia and a systemic inflammatory response. The exaggerated inflammatory response and the weakened adaptive immunity are responsible for the immunopathological complications of severe COVID-19 [6]. In fact, the hyperproduction of pro-inflammatory cytokines and chemokines called the “cytokine storm” (also known as “cytokine release syndrome”), is central to the pathogenesis, poor prognosis and fatal outcomes of COVID-19 [7].

The aberrant pro-inflammatory environment that accompanies the cytokine storm mainly originates from the crosstalk between epithelial and immune cells in COVID-19. Interestingly, recent findings have indicated that although abortive, SARS-CoV-2 infection of macrophages and dendritic cells cause cell activation and the subsequent release of multiple antiviral and pro-inflammatory cytokines and chemokines, aggravating the immunopathological picture and the cytokine storm [8].

While many studies have explored the clinical implications of the cytokine storm in COVID-19, the massive global vaccination campaigns have limited our understanding of the hypercytokinemia induced by SARS-CoV-2 infection. Therefore, as emphasized by recent papers, investigating the inflammatory microenvironment in non-vaccinated subjects remains crucial to better comprehend overlooked aspects of COVID-19 immunopathology [9], identify novel biomarkers of disease onset and progression, and develop more effective treatment strategies which could also be relevant for future viral pandemics.

Here, we present a detailed study of the levels and kinetics of 27 cytokines and chemokines in the sera of 21 SARS-CoV-2 positive patients during the first wave of the COVID-19 pandemic in Northern Italy.

## Methods

### Patient and sample collection

In this study, thirty patients admitted to the Istituto Clinico Città Studi (Milan, Italy) between March and April 2020 were included and divided into case (SARS-CoV-2 positive) and control (SARS-CoV-2 negative) groups, based on SARS-CoV-2 nasopharyngeal swab results. Specifically, the control group consisted of 9 patients, while the case group included 21 patients. The demographic and clinical characteristics of both groups are summarized in Table 1.

Clinical specimens were collected upon approval from the Local Ethical Committee and following the acquisition of signed informed consents (protocol 456_2020, approved in May 2020 by Fondazione Ca’ Granda, Ospedale Maggiore, Milan, Italy).

Blood samples were collected in tubes containing a clot activator and a gel to separate the serum from circulating cells. Samples were taken on the day of hospital admission (T0), on an intermediate day (T1, 18.4 ± 6.5 days), and on the last day of hospitalization (T2, 23.3 ± 16.0 days). T1 samples were available for 18 of the 21 patients in the case group.

### SARS-CoV-2 diagnosis

Positivity for SARS-CoV-2 infection was determined by nasopharyngeal swabs followed by molecular analysis of viral RNA. RNA was extracted from these samples using the Nucleospin RNA Virus Kit (Macherey-Nagel, Germany), following the manufacturer’s protocol. Quantification of viral RNA copy numbers in the cell supernatant were evaluated via specific qRT-PCR, targeting the N1 gene, as previously described [10], using the 7500 Gene Systems (Applied Biosystems, USA). A standard curve for quantification was generated using a plasmid containing the complete SARS-CoV-2 genome, using 10-fold serial dilutions ranging from 10^8^ to 10^0^.

### Cytokine/chemokine measurements and analysis

Serum samples were analyzed for the presence of 27 cytokines/chemokines (see Table S1 for the complete list) at T0, T1 and T2 using magnetic bead-based multiplex immunoassays (Bio-Plex^®^) (BIO-RAD Laboratories, Milano, Italy), following the manufacturer’s instructions. The selected multiplex panel was designed to include cytokines/chemokines relevant to both non-communicable and infectious diseases. This procedure employs Luminex Xmap technology using magnetic beads for multi-analyte profiling, as described previously [11].

The Th2/Th1 ratio was calculated by comparing the levels of Th2-related cytokines (IL-4, IL-6 or IL-10) with those of Th1-related pro-inflammatory cytokines (IFN-ɣ, TNF-α), as already reported [12].

### Detection of specific SARS-CoV-2 IgG antibodies in serum samples by ELISA

Semi-quantitative measurements of anti-SARS-CoV-2 IgG antibodies in sera were conducted using a commercial indirect ELISA colorimetric kit (ab275300, Abcam), according to the manufacturer’s instructions. The kit measures the presence of IgG recognising the S1-RBD domain of the Spike viral protein. After determining the validity of the Positive and Negative Controls relative to the Calibrator value, the values of the samples were compared to the Calibrator to generate a ratio. Ratios above or below the predefined cutoff indicated positive and negative samples, respectively.

### Statistical analysis

Statistics were determined using GraphPad Prism (version 9.0). The patients’ data were tested using a Shapiro–Wilk normality test, which determined the non-normal distribution of the data. Cytokine/chemokine values were analyzed under log10 (Log) transformation and compared between SARS-CoV-2-positive and SARS-CoV-2-negative patients by unpaired t test with or without the Welch’s correction. In addition, the effect of the pharmacological treatment on cytokine levels was analysed by paired t-test. One-way ANOVA and Tukey’s multiple comparison test were used to assess the kinetics of IL-6 and IP-10 in SARS-CoV-2 positive patients, as well as the changes in IgG titers throughout the study period. Correlations between IgG titers vs. viral load and cytokine/chemokine levels were assessed by Spearman’s test. Heat map analyses of the correlation coefficients between IgG and cytokine/chemokine levels were also conducted. Two-sided values of *p* less than 0.05 were considered statistically significant.

## Results

### Modulation of cytokine/chemokine levels in infected versus uninfected SARS-CoV-2 subjects

**Fig. 1:**
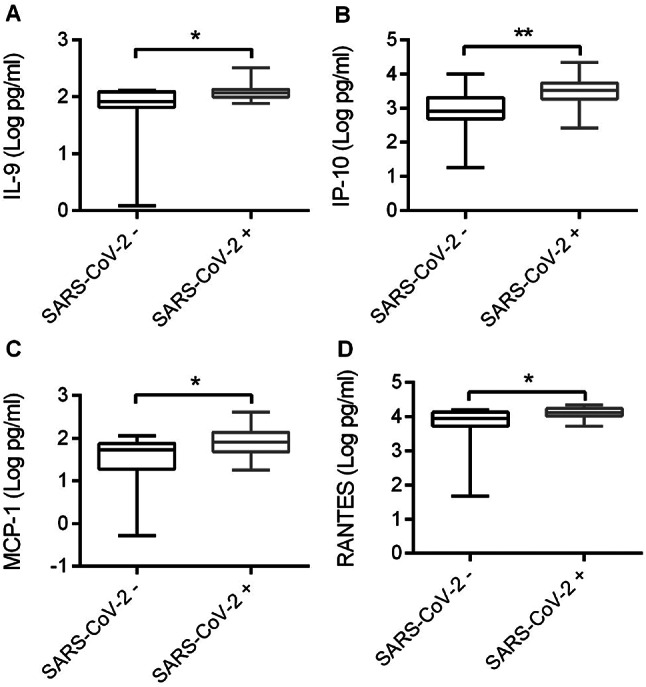
Cytokine and chemokine levels of SARS-CoV-2-positive and SARS-CoV-2-negative patients. Cytokines/chemokines (**A**: IL-9; **B**: IP-10; **C**: MCP-1; **D**: RANTES) were measured in serum samples by multiplex immunoassays and analyzed related to SARS-CoV-2 infection status. Data are presented as log10 (Log) of concentrations (picograms per milliliter, pg/ml) and shown as median with interquartile ranges. Statistical analyses were performed using an unpaired t test. *p < 0.05; **p < 0.01

The demographic and clinical characteristics of the patients are summarized in Table 1. Regarding the severity of respiratory symptomatology, two patients were admitted to intensive care unit, three received non-invasive ventilation (NIV), fourteen received conventional oxygen therapy, and two did not receive any respiratory support. A comparative analysis of the cytokine/chemokine serum profiles between SARS-CoV-2-positive and SARS-CoV-2-negative patients is detailed in Table S1. Figure 1 reports the levels of cytokines/chemokines which were significantly different in the two groups at T0. Notably, increased levels of IL-9 (*p* = 0.0305), IP-10 (*p* = 0.0069), MCP-1 (*p* = 0.0275) and RANTES (*p* = 0.0370) were observed in COVID-19 patients compared to SARS-CoV-2 negative subjects (Fig. 1A, B, C and D, respectively).

As reported in Table 1, there were 3 fatalities in the SARS-CoV-2 positive group. While this small number precluded formal statistical analyses, we observed that at T0 the levels of both IP-10 and MCP-1 in deceased patients were the highest recorded within the SARS-CoV-2-positive group (Fig. S1).

**Table 1:** Demographic and clinical characteristics of control subjects and COVID-19 patients (n.a., not applicable)

			Control subjects (negative SARS-CoV-2 swab)	COVID-19 patients (positive SARS-CoV-2 swab)
n			9	21
Sex assigned at birth (Female/Male)			5/4	10/11
Age at recruitment (mean ± SD)			70.7 ± 20.7	67.3 ± 17.1
Symptoms n (%)	Pneumonia		0 (0)	12 (57.1)
	Respiratory failure		1 (11.1)	9 (42.9)
	Gastrointestinal		0 (0)	7 (33.3)
	Others		8 (88.9)	0 (0)
Discharge type n (%)		Transferred	n.a.	13 (61.9)
		Ordinary	n.a.	5 (23.8)
		Deceased	n.a.	3 (14.3)
Early treatment (first 2–3 days post admission) n (%)		Azithromycin (AZ)	n.a.	3 (14.3)
		Hydroxychloroquine (HCQ)	n.a.	5 (23.8)
		AZ + HCQ	n.a.	5 (23.8)
		Other	n.a.	8 (38.1)

### Kinetics of cytokines/chemokines over time

**Fig. 2:**
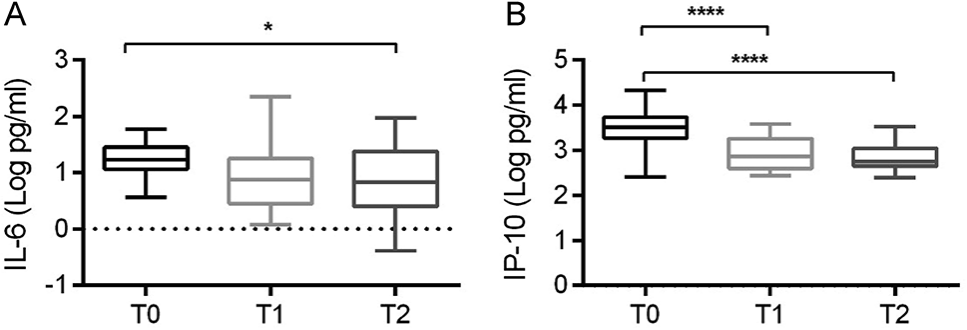
Kinetics of IL-6 (**A**) and IP-10 (**B**) in SARS-CoV-2 positive patients during hospitalization. The concentrations of cytokines/chemokines are represented as log10 (Log) of concentrations (picograms per milliliter, pg/ml). Statistical analyses were performed using one-way ANOVA and Tukey’s multiple comparison test. *p < 0.05; ****p < 0.001

To obtain longitudinal changes of cytokines/chemokines in the two groups, serum concentrations were quantified at three time points: upon hospital admission (T0), at an intermediate time (T1), and on the last day of hospitalization (T2). While most cytokines/chemokines did not show significant differences over time (Table S2), both IL-6 and IP-10 exhibited a significant decrease throughout the infection period (*p* = 0.0421 and *p* < 0.0001, respectively) (Fig. 2). It indicates a time-dependent modulation of IL-6 and IP-10 levels correlating with disease progression. The levels of cytokines/chemokines which were significantly different in SARS-CoV-2 positive and negative patients (IL-9, IP-10, MCP-1, RANTES) as well as those that decreased over time (IL-6, IP-10) were analyzed for correlation with the viral load in nasal swabs. However, no significant correlation was observed (Fig. S2).

### Effect of early pharmacological treatment on IP-10 and IL-6 plasma levels

**Fig. 3:**
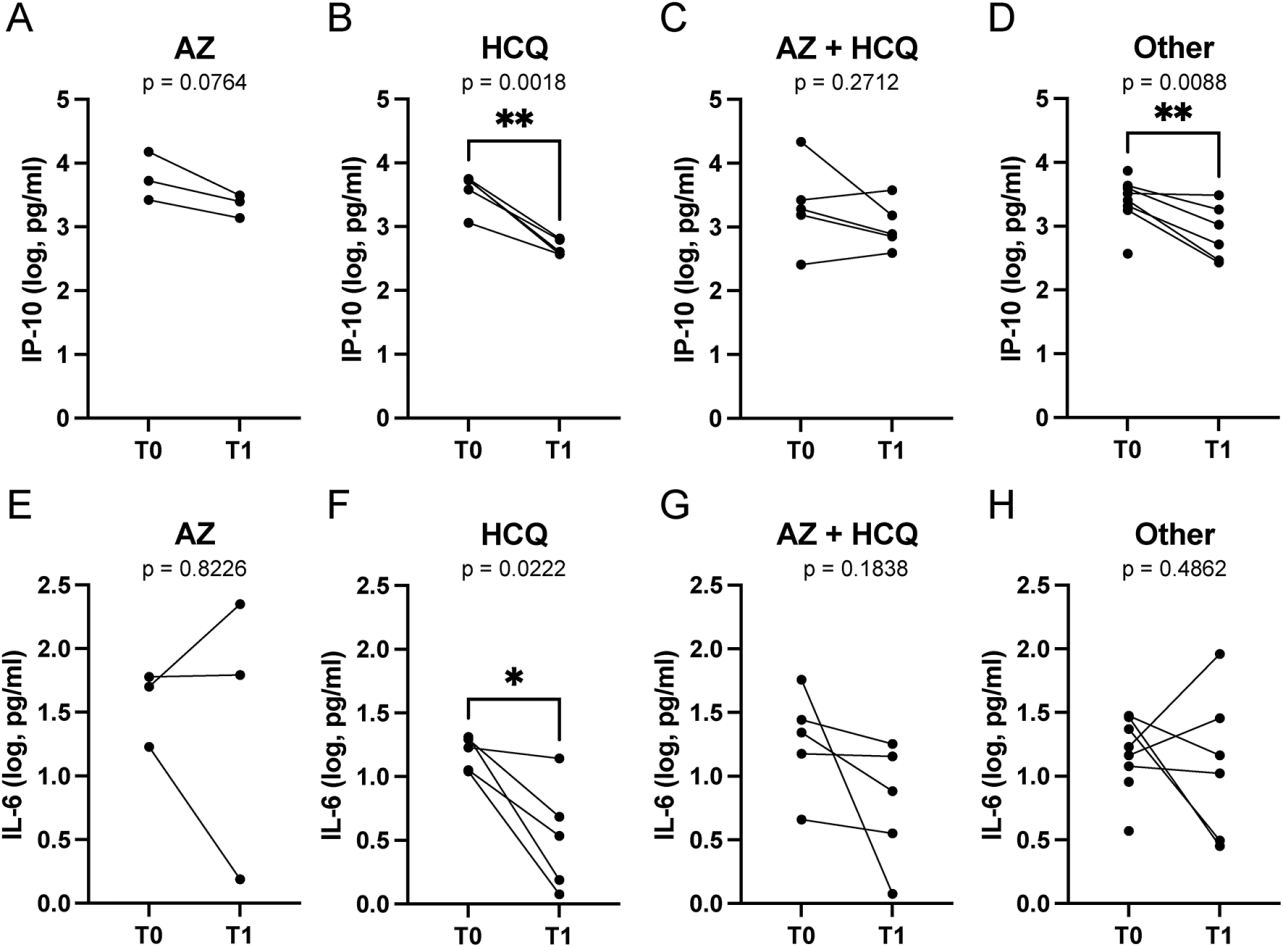
Effect of early treatment on IP-10 and IL-6 plasma levels between T0 and T1. **A-D)** IP-10 levels following treatment with azithromycin (AZ), hydroxychloroquine (HCQ), their combination (AZ + HCQ) and other treatments (Other), respectively. **E-H)** Variations in IL-6 serum levels post-treatment with AZ, HCQ, their combination and other therapeutic regimens, respectively. Concentrations of IL-6 and IP-10 are provided as log10 (Log) of concentrations (picograms per milliliter, pg/ml). Statistical analyses were performed using paired t test. Statistical significance is indicated above the graphs

We next reasoned that the administered pharmacological treatments (Table 1) may have influenced the observed decreasing trends in IL-6 and IP-10 levels over time. To investigate this, we analyzed IL-6 and IP-10 levels in SARS-CoV-2 positive individuals treated with azithromycin (AZ), hydroxychloroquine (HCQ), the combination of both drugs, or other therapeutic regimens (acetylsalicylic acid alone or in combination with lopinavir/ritonavir, ketorolac) (Fig. 3). As a standard of care, paracetamol was administered to most of the patients at different stages of their hospitalization period. Considering the lack of approved pharmacological therapies at the time, only early time points (2–3 days post-hospitalization), when patients received single therapeutic treatments, were further analyzed. These analyses revealed that HCQ and other treatments distinct from HCQ, AZ, or their combination (labelled as “Other” in Fig. 3) were associated with a significant decrease in IP-10 levels between T0 and T1 (Fig. 3B and D). Specifically, treatment with HCQ was correlated with a reduction in IL-6 levels (Fig. 3F).

### Correlation analysis of serum cytokine/chemokine levels and IgG titers in SARS-CoV-2 infected patients

**Fig. 4:**
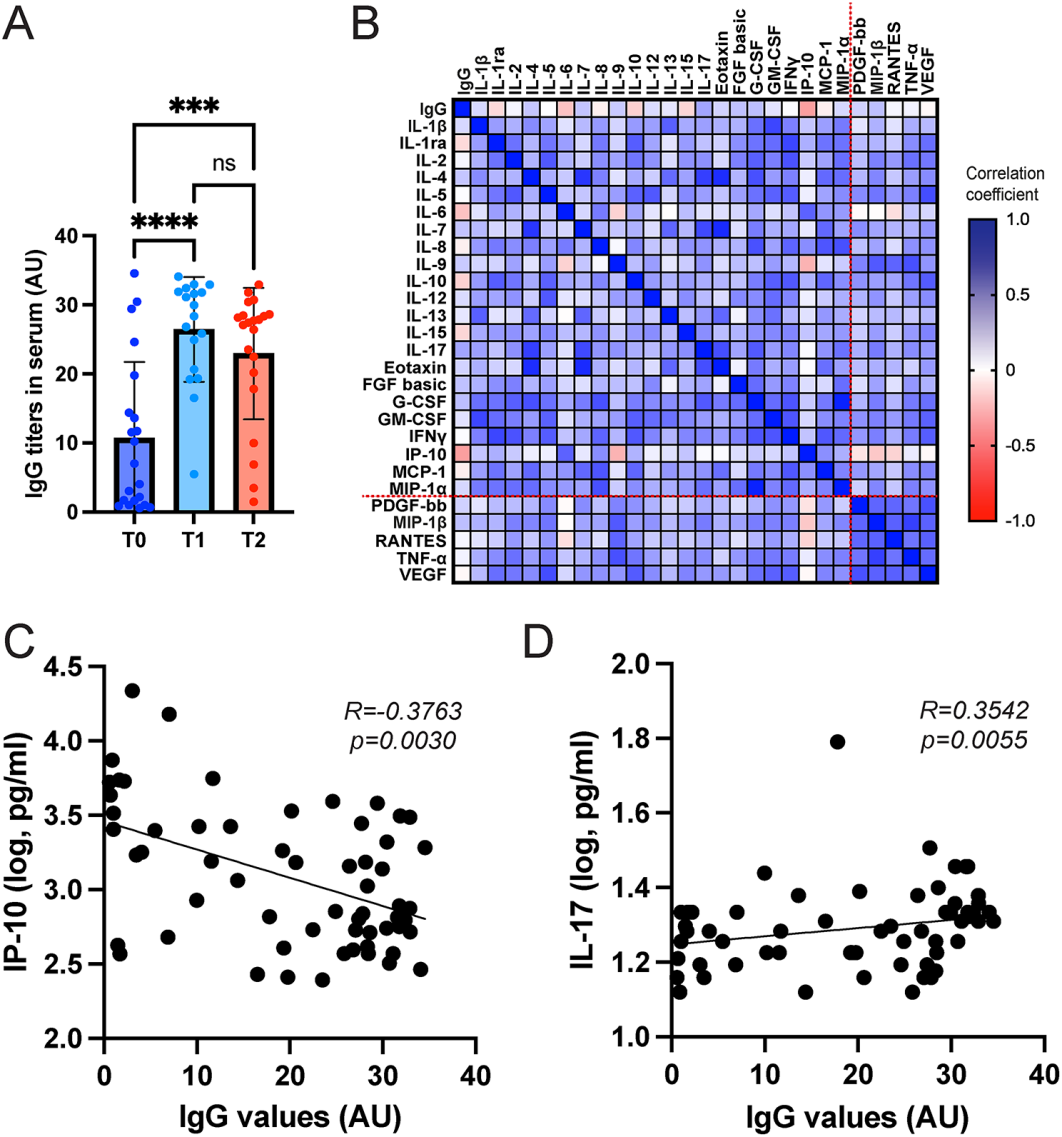
Correlations between serum cytokine/chemokine levels and anti-SARS-CoV-2 IgG antibodies. **(A)** IgG titers in SARS-CoV-2 infected patients at T0, T1 and T2. One-way ANOVA and Tukey’s multiple comparison test (****p < 0.001; ns, not significant). **(B)** Heat map representing Spearman’s correlation coefficients for cytokines/chemokines and IgG antibody titers. **C-D)** Scatter plots showing the correlations between IgG and IP-10 (C), and IL-17 (D). Each plot includes individual data points and a fitted line to indicate the correlation trend. R: correlation coefficient

We then asked whether the cytokine/chemokine levels found in the serum of SARS-CoV-2 infected patients might be correlated with the presence of specific antiviral IgG antibodies. Initially, we studied the trend of the IgG titers throughout T0, T1 and T2 and found a statistically significant increase from T0 to T1, while antibody levels remained unchanged from T1 until the last day of hospitalization (Fig. 4A). Next, we performed Spearman’s correlation analyses displayed as a heat map to study the associations between specific cytokines/chemokines and IgG levels in all samples, regardless of the collection time (Fig. 4B). We observed that PDGF-bb, MIP-1β, RANTES, TNF-α and VEGF formed a cluster of highly correlated cytokines (Fig. 4B, dotted red line). Interestingly, a negative and statistically significant correlation was found between IgG levels and IP-10 (Fig. 4C), whereas IL-17 showed a positive and significant association (Fig. 4D).

### Assessment of Th2/Th1 cytokine dynamics in COVID-19 patients

**Fig. 5:**
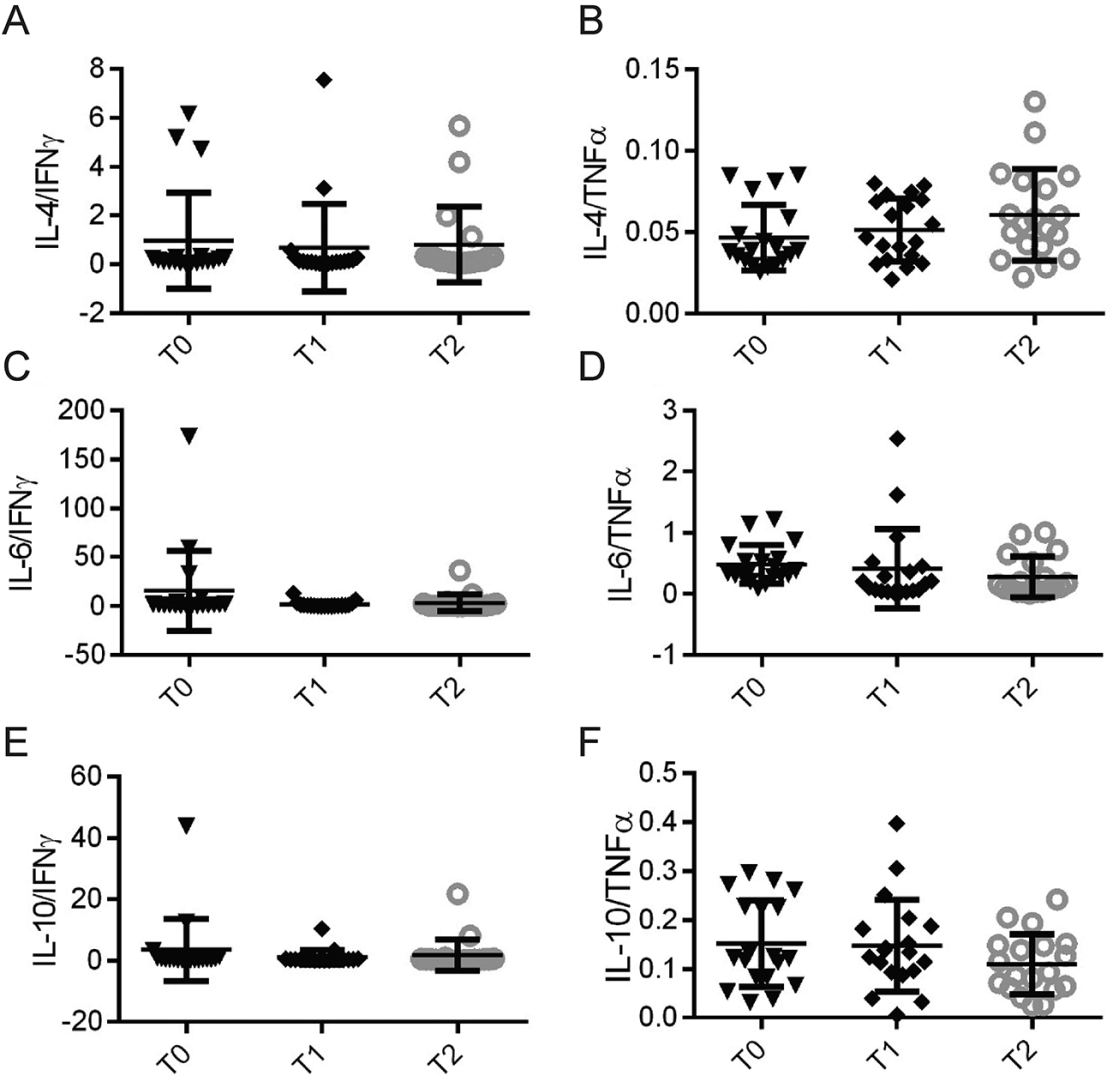
Dynamic Th2/Th1 ratios during infection period. Comparative Th2/Th1 ratios based on anti-inflammatory cytokines IL-4 (**A**, **B**), IL-6 (**C**, **D**) and IL-10 (**E**, **F**) vs. pro-inflammatory cytokines IFN-γ (**A**, **C**, **E**) and TNF-α (**B**, **D**, **F**). Box-and-whisker plots display the median and interquartile ranges

T-helper cells (Th) within the adaptive immune system play a crucial role against viral infections [13]. In COVID-19, disease severity and mortality have been linked to an imbalance in Th2/Th1 cytokine levels [12]. Thus, we aimed to further assess the Th2/Th1 cytokine levels in the sera of our cohort of COVID-19 patients over the course of their infection (Fig. 5). We observed that the ratios of Th2 cytokines (IL-4, IL-6, and IL-10) to IFN-γ remained constant over time (Fig. 5A, C, E). There was an increase in the IL-4/TNF-α ratio from the initial to the final time point (Fig. 4B), whereas both the IL-6/TNF-α and IL-10/TNF-α ratios exhibited a decrease (Fig. 5D and F). However, these changes did not reach statistical significance.

## Discussion

Several studies have reported elevated cytokine/chemokine levels and other circulating inflammatory mediators in the serum of COVID-19 patients, thereby intensifying the disease’s complexity [14]. This hyper-inflammatory response or “cytokine storm” can lead to widespread tissue damage, systemic inflammation, and potentially fatal organ dysfunction, significantly contributing to the severity and mortality of COVID-19 [15]. However, there is a high variability across studies in the specific cytokines/chemokines involved, often reflecting differences in disease characteristics, demographic factors, and clinical settings. In this study, we analyzed the levels and the kinetics of 27 cytokines/chemokines in patients primarily infected during the pandemic’s first wave, who were unexposed to vaccines or established treatments, and compared these to uninfected individuals. The aim was to uncover novel insights into the initial immune response against SARS-CoV-2 infection. This selected panel of cytokines and chemokines is a reliable collection of markers involved in different diseases including infectious diseases.

We show that the serum of SARS-CoV-2-positive patients presented higher levels of IL-9, RANTES, MCP-1 and IP-10 compared to negative individuals. MCP-1/CCL2 and RANTES/CCL5 belong to the CC family of chemokines, which regulate the migration of monocytes and T lymphocytes. Here, the elevated serum levels of both chemokines are in agreement with published reports [16, 17]. The expression of CC chemokines, rather than CXC chemokines, is often associated with viral infections. In particular, RANTES/CCL5 seems to be almost invariably enhanced during viral infections [18]. Despite their role in antiviral activity, whether these chemokines also contribute to the pathogenesis of the disease is still unclear. Consistent with previous studies, IP-10/CXCL10 was elevated in the serum of individuals suffering from COVID-19 disease [19], which aligns with its known association with acute respiratory infections [20] and disease severity [19, 21, 22]. Interestingly, a positive correlation between IP-10 levels and mortality was also observed in our cohort [23], with deceased patients also showing increased levels. Our findings also indicated higher IL-9 levels in COVID-19 patients. This is different from other studies where the levels of this cytokine were not different from that of controls [24, 25]. This discrepancy highlights the cytokine complex role. IL-9 is produced by CD4 + T cells, in particular Th2 cells, and is associated with allergic asthma. In addition, high levels of IL-9 are also present in bronchial secretions from infants with respiratory syncytial viral bronchiolitis [26].

By performing longitudinal analyses, a time-dependent reduction of IL-6 and IP-10 levels was observed. Since most of the patients recovered, these results are in line with previous studies demonstrating an association between IL-6 and IP-10 with disease progression [16, 27]. Indeed, IL-6 is recognized as a crucial mediator in the cytokine response to COVID-19 infection. Elevated levels of IL-6 are a significant indicator of the severity of COVID-19 and are closely associated with the increased mortality rate observed in patients [27]. Furthermore, plasma levels of IL-6 have been also associated with morbidity and mortality in patients with acute lung injury [28]. Despite the lack of differences observed regarding IL-6 as a biomarker for COVID-19 severity, our data are in agreement with the key role of both inflammatory factors in mediating the acute immune response.

Current evidence indicates that increased clinical severity in COVID-19 is often accompanied by elevated antibody levels [29]. However, the relationship between plasma IgG antibody titers and specific cytokine/chemokine levels is not completely understood. Our analysis revealed significant correlations between IgG levels and specific cytokines/chemokines. Specifically, IgG levels negatively correlated with IP-10, while a positive association was observed with IL-17. These findings are partially in line with previous studies, where negative associations with both IP-10 and G-CSF were reported [30]. Regarding IL-17, it has been suggested that the severity of COVID-19 is closely associated with IL-17-driven inflammation. Patients with COVID-19 who required intensive care unit (ICU) treatment exhibited higher levels of Th17 cells and more severe clinical symptoms compared to those not in the ICU, likely due to the overproduction of IL-17 and related cytokines [31]. Indeed, IL-17 blockade has been proposed as a viable therapeutic option to alleviate COVID-19 severity and reduce mortality related to acute respiratory distress syndrome (ARDS) [32]. Additionally, production of anti-Spike antibodies has been associated with virus neutralization [33, 34]. Thus, it is plausible that the detected IgG antibodies in our cohort of patients possess high neutralizing activities, too. This hypothesis, however, remains to be investigated, especially because the humoral response alone may not be sufficient to clear the infection.

Considering the effect of drug treatment, hydroxychloroquine (HCQ), known for its anti-inflammatory properties, significantly reduced IL-6 levels, an effect already observed in patients affected by systemic lupus erythematosus (SLE), a chronic autoimmune disease [35, 36]. Analogously, IP-10 was found to be reduced in SLE patients after HCQ treatment [37]. However, in the absence of an untreated group for comparison and due to the small sample size, a normal decrease of IL-6 and IP-10 levels cannot be completely ruled out. Regarding published reports, the WHO Solidarity Trial Consortium found that HCQ regimen had little or no effect on hospitalized COVID-19 patients, as indicated by overall mortality, initiation of ventilation, and duration of hospital stay [38]. In addition, Willis and colleagues described unaltered IL-6 and IP-10 levels upon HCQ treatment [39]. In the light of these findings and despite the small sample size our cohort, our results might indicate variability in HCQ’s immunomodulatory effects, further emphasizing the need for additional investigations on the relationship between cytokines/chemokines and COVID-19 immunomodulating pharmacological treatments.

The type of T helper (Th) cell response is critical in influencing the progression and outcome of COVID-19 [40]. In particular, the two major subtypes of Th cells, Th1 and Th2, coordinate distinct immune responses to infections. Th1 cells produce cytokines such as IL-2 and IFN-γ that coordinate the cell-mediated immune response, while Th2 cells secrete cytokines like IL-4 and IL-6, which support the humoral response [41]. Notably, IL-6, often produced by Th2 cells, has complex roles and can also be involved in pro-inflammatory processes [42]. An imbalance in the Th2/Th1 immune response has been implicated in the immunopathology of COVID-19, though this concept is still controversial [43]. In fact, the prognosis tends to worsen and the risk of mortality increases when the Th2 response predominates over the Th1 response [42]. Here, we observed that the ratios of Th2 to Th1 cytokines, specifically anti-inflammatory cytokines (IL-4, IL-6, and IL-10) relative to IFN-γ, remained stable throughout the infection. Notably, we observed an increase in the IL-4/TNF-α ratio and a decrease in both IL-6/TNF-α and IL-10/TNF-α ratios. This contrasts with findings from other studies, where severe COVID-19 cases showed a higher IL-6/IFN-γ ratio than milder cases, suggesting a stronger cytokine storm leading to lung damage [44]. Moreover, a positive correlation between the TNF-α/IL-10 ratio and both respiratory failure and disease severity has been documented [14]. These results highlight the intricate dynamics between Th cell responses, cytokines and the cytokine storm in the progression and immunopathology of COVID-19 [45].

## Conclusions

In conclusion, despite the small cohort size, our research contributes to the growing body of knowledge on the primary immune response to SARS-CoV-2, particularly in the context of the early pandemic phase, when neither approved vaccines nor specific antiviral treatments were available. It further highlights the necessity for additional research to investigate the complex interplay between cytokines/chemokines, antibody responses, and clinical outcomes in COVID-19. The insights gained from this study not only enhance our understanding of the disease immunopathology but also might pave the way for future research in developing targeted therapeutic strategies.

### Electronic supplementary material

Below is the link to the electronic supplementary material.

Supplementary Material 1

## Data Availability

The datasets supporting the conclusions of this article are included within the article and its additional file. Study protocols, including laboratory protocols, will be available upon request.

## Abbreviations

COVID-19: Coronavirus disease 2019
IL-9: Interleukin-9
IL-6: Interleukin-6
IL-17: Interleukin-17
IL-4: Interleukin-4
IL-10: Interleukin-10
IL-6: Interleukin-6
CFR: Cumulative case fatality rate
ACE-2: Angiotensin-converting enzyme-related carboxypeptidase 2
AZ: Azithromycin
HCQ: Hydroxychloroquine
Th1: T-helper cells type 1
Th2: T-helper cells type 2
ICU: Intensive care unit
ARDS: Acute respiratory distress syndrome
SLE: Systemic lupus erythematosus
