# Supplementary material for: Multiplex array analysis of circulating cytokines and chemokines in COVID-19 patients during the first wave of the SARS-CoV-2 pandemic in Milan, Italy
Source: BMC Immunol. 2024 Jul 26;25:49. doi: 10.1186/s12865-024-00641-z (PMC11282750; doi:10.1186/s12865-024-00641-z)
Supplement: Supplementary file 1 — Supplementary Material 1 [file 12865_2024_641_MOESM1_ESM.docx]

**Multiplex array analysis of circulating cytokines and chemokines in COVID-19 patients during the first wave of the SARS-CoV-2 pandemic in Milan, Italy**

Estefanía Calvo-Alvarez^1^, Sarah D’Alessandro^1,^*, Nunzia Zanotta^2^, Nicoletta Basilico^3^, Silvia Parapini^4^, Lucia Signorini^3^, Federica Perego^3^, Kevin Maina^3^, Pasquale Ferrante^3^, Annalisa Modenese^5^, Pierluigi Pizzocri^5^, Andrea Ronsivalle^5^, Serena Delbue^3^, Manola Comar^2^

^1^ Department of Pharmacological and Biomedical Sciences, Via Carlo Pascal, 36, Università degli Studi di Milano, 20133 Milan, Italy.

^2^ Department of Advanced Translational Microbiology, Institute for Maternal and Child Health—IRCCS Burlo Garofolo, Via dell’Istria, 65, Trieste, 34137, Italy.

^3^ Department of Biomedical, Surgical and Dental Sciences, Via Carlo Pascal, 36, Università degli Studi di Milano, 20133 Milan, Italy.

^4^ Department of Biomedical Sciences for Health, Università degli Studi di Milano, Via Carlo Pascal, 36, 20133 Milan, Italy.

^5^ Istituto Clinico Città Studi, Milano, Via Ampere 47, 20133 Milano, Italy

**Table S1.** Concentrations of immune mediators in serum of SARS-CoV-2 positive (n=21) versus negative (n=9) patients. Data are provided as median and quartiles ranges (pg/mL).

| **Immune effector cell** | **Immune mediator** | **Type of mediator** | **SARS-CoV-2 negative** | **SARS-CoV-2 positive** | **p-value*** |
| --- | --- | --- | --- | --- | --- |
| Th1 | IFN-γ | Cytokine | 15.15 (2.34-25.05) | 21.21 (6.66-32.58) | 0.4685 |
|  | IP-10 (CXCL10) | Chemokine | 2026 (487-2087) | 4669 (1849-5393) | 0.0138 |
|  | TNF-α | Cytokine | 57.43 (47.41-67.10) | 52.98 (40.27-62.67) | 0.5811 |
| Th2 | IL-4 | Cytokine | 2.95 (2.34-4.08) | 2.33 (1.79- 2.71) | 0.8224 |
|  | IL-5 | Cytokine | 2.57 (0.94-4.87) | 6.26 (0.94-10.55) | 0.4574 |
|  | IL-9 | Cytokine | 82.72 (65.44-122.0) | 125.01 (99.00-134.27) | 0.0268 |
|  | IL-13 | Cytokine | 5.12 (2.79-6.67) | 4.21 (3.48-5.28) | 0.6501 |
|  | Eotaxin (CCL11) | Chemokine | 74.72 (32.02-112.80) | 100.50 (63.19-126.11) | 0.0880 |
| Th17 | IL-6 | Cytokine | 18.39 (4.32-32.52) | 22.44 (11.60-28.41) | 0.1713 |
|  | IL-8 (CXCL8) | Cytokine | 31.43 (10.56-44.85) | 20.55 (16.25-24.22) | 0.8270 |
|  | IL-17 | Cytokine | 26.09 (15.61-20.68) | 18.81 (15.91-21.57) | 0.9372 |
| T reg | IL-10 | Cytokine | 28.28 (1.26-13.37) | 7.91 (4.17-11.57) | 0.5810 |
| Broad spectrum | IL-1β | Cytokine | 1.84 (1.45-2.49) | 1.84 (1.38- 2.00) | 0.6651 |
|  | IL-1ra | Cytokine | 1306 (556-2157) | 1128 (398-1627) | 0.4951 |
|  | IL-2 | Cytokine | 5.59 (5.02-6.63) | 6.26 (5.02-7.75) | 0.7104 |
|  | IL-7 | Cytokine | 30.33 (16.20-41.98) | 34.51 (23.67-43.98) | 0.1932 |
|  | IL-12(p70) (75) | Cytokine | 3.51 (2.08-2.08) | 4,11 (2.08-2.08) | 0.8777 |
|  | IL-15 |  | 3.17 (3.17-3.17) | 24.61 (3.17-3.17) | 0.3859 |
|  | FGF basic | Trophic factor | 36.85 (37.23-43.66) | 50.09 (35.61-40.37) | 0.1853 |
|  | G-CSF | Trophic factor | 281.3 (148.7-378.3) | 223.3 (147.0-246.6) | 0.7462 |
|  | GM-CSF | Trophic factor | 1.90 (0.59-.50) | 2.27 (0.89-3.26) | 0.5401 |
|  | MCP-1(MCAF) (53) |  | 51.95 (20.97-74.84) | 103.50 (48.12-137.18) | 0.0372 |
|  | MIP-1α (CCL3) | Chemokine | 5.73 (2.23-7.68) | 3.53 (2.34-3.57) | 0.4172 |
|  | PDGF-bb | Trophic factor | 3074 (1467-4740) | 3726 (1990-5225) | 0.3760 |
|  | MIP-1β (CCL4) | Chemokine | 69.53 (51.75-98.24) | 77.93 (58.49-89.41) | 0.1259 |
|  | RANTES (CCL5) | Chemokine | 8672 (5284-13207) | 13480 (10405-17024) | 0.0297 |
|  | VEGF | Trophic factor | 34.26 (1.20-69.26) | 85.88 (1.20-179.50) | 0.2361 |

*p-values were calculated on the log10 (Log) data

**Table S2.** Concentrations of immune mediators in serum of SARS-CoV-2 positive (n=21) patients. Data are provided as median and quartiles ranges (pg/mL).

| **Immune mediator** | **T0** | **T1** | **T2** |
| --- | --- | --- | --- |
| IL-1β | 1.78 (1.36-1.90) | 1.82 (1.45-2.22) | 2.23 (1.45-2.44) |
| IL-1ra | 1012 (378.9-1446) | 1148 (475.5-1416) | 1835 (387.4-922.9) |
| IL-2 | 6.06 (5.02-6.86) | 6.50 (5.02-6.86) | 6.44 (5.02-7.75) |
| IL-4 | 2.31 (1.71-2.64) | 2.63 (1.71-3.57) | 3.48 (1.71-4.32) |
| IL-5 | 6.13 (0.94-8.12) | 6.95 (0.94-9.38) | 6.11 (0.94-9.38) |
| IL-6 | 22.69 (13.92-28.06) | 15.75 (2.50-15.41) | 16.28 (1.87-24.08) |
| IL-7 | 34.65 (25.61-43.42) | 34.84 (28.13-43.98) | 35.96 (23.03-47.85) |
| IL-8 | 20.72 (16.04-26.42) | 23.66 (12.13-31.47) | 68.61 (11.13-39.94) |
| IL-9 | 124.5 (99.00-132.7) | 125.5 (105.2-137.8) | 118.6 (99.00-133.8) |
| IL-10 | 7.93 (4.17-12.30) | 7.88 (5.30-11.57) | 6.03 (2.65-8.27) |
| IL-12(p70) (75) | 3.31 (2.08-2.08) | 2.89 (2.08-3.67) | 3.57 (2.08-5.09) |
| IL-13 | 4.22 (3.48-5.44) | 4.32 (3.14-4.80) | 5.01 (3.48-6.37) |
| IL-15 | 26.87 (3.17-3.17) | 3.17 (3.17-3.17) | 63.84 (3.17-98.98) |
| IL-17 | 18.96 (16.21-21.57) | 19.96 (18.00-21.57) | 22.65 (15.60-25.10) |
| Eotaxin | 100.50 (63.19-126.1) | 111.5 (66.82-183.8) | 122.7 (64.74-181.1) |
| FGF basic | 51.28 (34.78-39.61) | 51.93 (34.78-46.77) | 42.63 (34.78-44.01) |
| G-CSF | 215.7 (143.3-238.2) | 267.9 (184.3-295.0) | 263.1 (145.4-343.1) |
| GM-CSF | 2.22 (0.64-3.43) | 2.76 (1.98-3.43) | 3.12 (0.49-4.57) |
| IFN-γ | 20.60 (7.27-29.56) | 26.63 (9.86-32.07) | 31.54 (5.54-48.26) |
| IP-10 | 4751 (1918-5347) | 1243 (393.4-1824) | 906.9 (479.8-848.1) |
| MCP-1(MCAF) (53) | 104.1 (43.9-140.2) | 123.0 (31.55-127.6) | 138.1 (34.41-168.2) |
| MIP-1α | 3.34 (2.25-3.55) | 4.36 (2.29-5.05) | 3.90 (2.07-4.68) |
| PDGF-bb | 3645 (2031-5200) | 4578 (2567-5894) | 3765 (2172-4638) |
| MIP-1β | 78.83 (59.41-89.25) | 86.80 (68.97-92.93) | 85.80 (78.72-92.12) |
| RANTES | 13105 (10100-16604) | 13011 (10325-16145) | 12915 (10989-14716) |
| TNF-α | 51.93 (39.51-58.97) | 51.85 (41.02-57.48) | 55.26 (41.78-61.93) |
| VEGF | 75.41 (1.20-179.5) | 118.2 (1.20-201.6) | 105.6 (1.20-175.0) |

T0 = first day of hospital admission; T1 = intermediate time; T2 = last day of hospital admission.


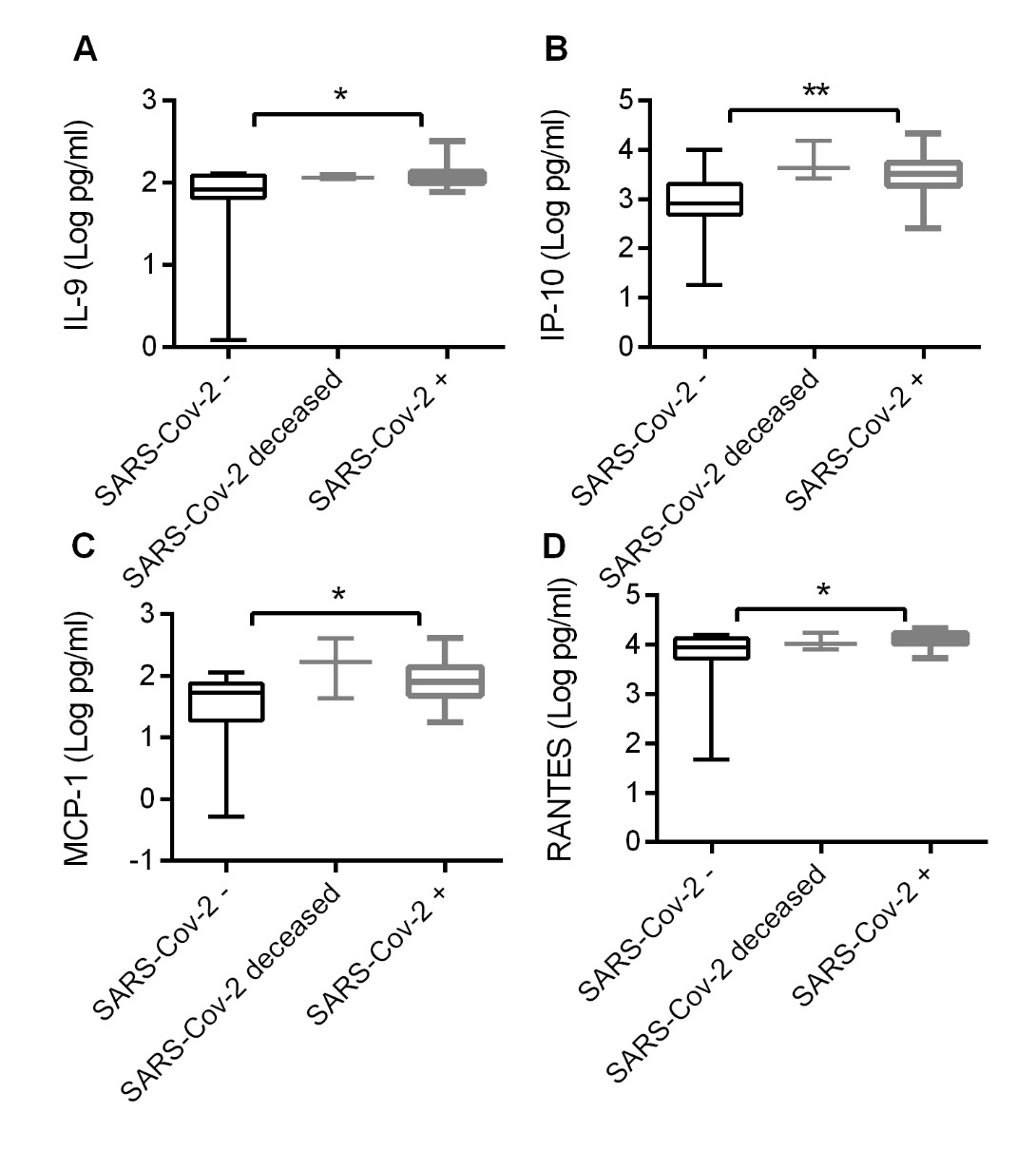


**Figure S1.** Cytokine and chemokine levels in SARS-CoV-2-negative subjects, deceased SARS-CoV-2-positive patients, and SARS-CoV-2-positive patients who recovered. Cytokines and chemokines (A: IL-9; B: IP-10; C: MCP-1; D: RANTES) were measured in serum samples using multiplex immunoassays and analyzed in relation to SARS-CoV-2 infection status. Data are presented as log10 (Log) of concentrations (picograms per milliliter, pg/ml). Statistical analyses were performed using an unpaired t-test. *p<0.05; **p<0.01.

**Figure S2.** Correlation analysis between viral load and serum cytokines/chemokines levels. Each scatter plot represents individual patient data points, assessing the relationship between viral load detected in nasal swabs and serum levels of specific cytokines/chemokines (A: IL-9; B: IP-10; C: MCP-1; D: RANTES; E: IL-6). The Spearman correlation coefficient (R) and the associated p-value are displayed on each graph, with a fitted line illustrating the correlation trend. No significant correlation was observed across the cytokines/chemokines tested.

**Table S3.** Anti-Spike IgG levels in the sera of SARS-CoV-2 positive patients by semi-quantitative ELISA. Data are provided as median with quartile ranges.

| **SARS-CoV-2 positive patients**  **IgG (ratio)*** | | |
| --- | --- | --- |
| **T0 (n=21)^#^** | **T1 (n=18)^#^** | **T2 (n=21)^#^** |
| 10.6713 (0.5895-34.5606) | 26.4332 (5.4797-34.0751) | 22.9372 (1.4682-32.9017) |

*Measurements were conducted using a commercial indirect ELISA colorimetric kit (ab275300, Abcam), according to the manufacturer’s instructions. After determining the validity of the Positive and Negative Controls relative to the Calibrator value, the values of the samples were compared to the Calibrator to generate a ratio. Ratios equal to or greater than 0.9 were considered positive, while ratios of 0.8 or less were considered negative. Ratios between 0.8 and 0.9 were classified as equivocal, indicating indeterminate results.

#T0 = first day of hospital admission; T1 = intermediate time; T2 = last day of hospital admission.

**Figure S3.** Correlations between serum cytokines/chemokines and anti-SARS-CoV-2 IgG antibodies during distinct hospitalization periods. The samples are divided into early (from day 1 to 14, A-I) and late (from day 15 to 7 weeks, J-L) response periods. Heat maps represent Spearman’s correlation coefficients for cytokines/chemokines and IgG antibody titers in early (A) and late (J) response samples. Scatter plots show the correlations between IgG levels and individual cytokines/chemokines in early (B-I) and late response samples (K-L). Each plot includes individual data points with a fitted line indicating the correlation trend. Only significant correlations are shown (p < 0.05). R: correlation coefficient.
